# Microfinance and Peer Health Leadership Intervention Implementation for Men in Dar es Salaam, Tanzania: A Qualitative Assessment of Perceived Economic and Health Outcomes

**DOI:** 10.1177/1557988320936892

**Published:** 2020-07-04

**Authors:** Frank Mhando, Kathryn Dovel, Larissa Jennings Mayo-Wilson, Deusdedit Rwehumbiza, Noah Thompson, Ucheoma Nwaozuru, Abubakar Rehani, Juliet Iwelunmor, LaRon E. Nelson, Donaldson Fadael Conserve

**Affiliations:** 1 Department of Geography, University of Dar es Salaam, Dar es Salaam, Tanzania; 2 Division of Infectious Diseases, David Geffen School of Medicine, University of California Los Angeles (UCLA), Los Angeles, USA; 3 Department of Applied Health Science, Indiana University School of Public Health, Bloomington, IN, USA; 4 Department of International Health, Johns Hopkins University School of Public Health, Baltimore, MD, USA; 5 Department of Health Promotion, Education, and Behavior, University of South Carolina, Columbia, SC, USA; 6 Department of Behavioral Science and Health Education, Saint Louis University, Saint Louis, MO, USA; 7 Marie Stopes Tanzania, Dar es Salaam, Tanzania; 8 Yale University School of Nursing, West Haven, CT, USA

**Keywords:** Men, economic, microfinance, income, HIV testing, health-seeking behavior, intervention, qualitative, Tanzania

## Abstract

Men in sub-Saharan Africa continue to experience health disparities that are exacerbated by low employment. This study qualitatively assessed men’s perceptions of the economic and health-care-seeking effects of participation in an integrated microfinance and peer health leadership intervention on violence and HIV risk reduction in Tanzania. Three focus group discussions with 27 men, aged 20 to 44 years, examined the perceived effects on income generation, employability, mental health, and uptake of HIV and related health services. All discussions were recorded, transcribed, and analyzed using deductive and inductive coding methods. Men reported that the benefits of the intervention included increased employability and income-earning activities due to greater access to entrepreneurial training, low-interest microfinancing, and male-oriented group supports to start or strengthen their businesses. Increased wages through business or other forms of employment were also attributed to men’s lower anxiety and distress as financial providers for their families. However, men indicated that apart from the uptake of free HIV testing services, there was limited change in overall health-care-seeking behavior given the high clinic fees and lost time to earn income when attending routine health visits. Men recommended that future microfinance and health promotion interventions provide larger loan amounts, less frequent repayment intervals, and access to health and social insurance. Microfinance and peer health leadership interventions may help to address economic and health disparities in poor, urban men. Efforts are needed to assist lower income men in accessing financial tools as well as fee-based preventive and health-care services.

Men continue to experience higher mortality and lower life expectancy than women, in part due to limited use of health services, increased risk of injury, alcohol abuse, and mental illness (Baker et al., 2014; Baker & Shand, 2017; Dicker et al., 2018; Teo et al., 2016). Poverty exacerbates these health disparities. Men of lower socio-economic status in sub-Saharan Africa are at increased risk compared to their wealthier male counterparts for perceived stress and mental health illnesses such as depression and anxiety (Hamad et al., 2008), HIV acquisition, development of noncommunicable diseases (NCDs; Adedoyin et al., 2005), and general poor use of health services (Teo et al., 2017). Engaging men of lower socio-economic status in health services is crucial for promoting men’s health and curbing HIV epidemics in the region (Amidou et al., 2019; Bigna & Noubiap, 2019; Ciccacci et al., 2019; Mills et al., 2012). Immediate strategies are needed to address poverty among men and their families in sub-Saharan Africa to promote health services uptake, improve men’s general health, and reduce economic stressors associated with mental health (Teo et al., 2017).

Economic empowerment programs such as microfinance offer a promising strategy to holistically address economic stressors, disparities in health services uptake, and health outcomes faced by poor men. For example, access to credit through loans was associated with a reduction in depressive symptoms among men in South Africa (Fernald et al., 2008). To date most microfinance studies have focused on women and a positive association between microfinance participation and health-seeking behaviors (i.e., antenatal care) has been reported among women (Bhuiya et al., 2018; Leite et al., 2019; Mergenova et al., 2019). Only one randomized clinical trial targeting men has included microfinance as a part of combined microfinance and peer health leadership intervention to increase HIV testing among men (Kajula et al., 2016). The study reported higher levels of past-year HIV testing at 30-months follow-up compared to participants in the control group (Maman et al., 2020). While the intervention increased uptake of HIV testing, it is crucial to further explore how the intervention may have influenced men’s health and general health–seeking behaviors.

Combining health interventions with microfinance programs may have benefits for men’s general health. Entrepreneurial activities among men may reduce mental health concerns, such as anxiety and depression, that are associated with insecure and insufficient income (Fernald et al., 2008; Hamad et al., 2008). Throughout the region men are socialized to be financial providers for their families and may become stressed or depressed when they are unable to provide financially (Barker & Ricardo, 2005; Hunter, 2006; Silberschmidt, 2001). In Tanzania, 22% of men reported clinically significant symptoms of depression and 20% reported clinically significant symptoms of anxiety (Hill et al., 2018). Men with higher anxiety and depression scores were less likely to use condoms and more likely to have multiple sexual partners compared to men with lower anxiety or lower depression scores (Hill et al., 2017). Men in Tanzania described perceived associations between economic strain and their social and mental well-being and requested that employment and related microfinance intervention be incorporated into health interventions (Yamanis et al., 2010). Microfinance interventions may also help reduce economic stressors for men who are sole financial providers for their families and help them generate the income needed to cover the costs associated with seeking health services. Yet there have been limited considerations given to address economic challenges that affect men’s social and mental well-being and that may hinder men’s uptake of health services (Hamad et al., 2008; Teo et al., 2017).

This study used qualitative data to assess the benefits and challenges related to participating in a combined microfinance and peer health leadership intervention for men in Tanzania, including a more nuanced contextual understanding of the intervention’s perceived effects on men’s income and financial security and their mental health in relation to their role as providers for their families. Men’s general health–seeking behaviors after having participated in the combined intervention recommendations for future microfinance and peer health leadership interventions were also examined.

## Methods

### Design and Setting

A descriptive qualitative study was conducted with a subsample of men who participated in the intervention arm of the Vijana Vijiweni II (VVII) project. VVII was a 30-month cluster randomized clinical trial (cRCT) designed to assess the efficacy of a combined microfinance and peer health leadership intervention on HIV and gender-based violence prevention among men in four wards (i.e., Manzese, Mabibo, Tandale, Mwananyamala) within the Kinondoni District of Dar es Salaam, Tanzania (Kajula et al., 2016). The protocol and outcomes for VVII are described in detail elsewhere (Kajula et al., 2016; Yamanis et al., 2010). The qualitative study was undertaken in accordance with the Standards for Reporting Qualitative Research (O’Brien et al., 2014). This study is based on pragmatism as a research paradigm (Savin-Baden & Howell-Major, 2013) with a focus on economic empowerment of men experiencing poverty in sub-Saharan Africa. That is, the study objectives are practical and related to effecting a change—ultimately increasing life expectancy rates for men in this region. Pragmatism as a paradigm focuses on the “practical effects of what is believed and, in particular the usefulness of these effects,” in other words, on the workability of ideas—what works in practice (Savin-Baden & Howell Major, 2013, p. 60).

### Intervention Description

The intervention consisted of microfinance and peer health leadership strategies implemented over a 30-month period. The microfinance component was led by Youth Self Employment Opportunity (YOSEFO), a local microfinance institution (MFI), and included a 5-day business and finance training focusing on entrepreneurship, business development and expansion, and three loans ($100, $185, and $285) followed by booster sessions held at 6-month intervals for borrowers (Balvanz et al., 2019). In order to qualify for the loans, intervention camp members had to complete the following requirements: (a) attend the 5-day training, (b) complete the baseline survey, (c) deposit $5 in savings, (d) pay $5 loan fee, (e) document physical collateral against the loan, (f) form a loan solidarity group containing up to five members, and (g) obtain loan application approval by group and camp leaders (Balvanz et al., 2019). Among 621 intervention participants, 162 men from 22 out of the 30 camps took a loan (Balvanz et al., 2019). For the peer health leadership component, 170 members from the intervention camps were nominated by their peers and they attended a 5-day training to become popular opinion leaders referred to as community health leaders (CHLs; Kajula et al., 2019). The training focused on leadership, gender-based violence and power, HIV and condom myths, safe sexual practices, communication, and effective messaging. After the training, CHLs were then encouraged to engage in conversations with their peers to promote HIV testing, reduce sexual risk behaviors, and reduce gender-based violence. CHLs attended a 1-day booster session to discuss successes and challenges and were provided with journals to keep record of the number and type of conversations.

### Sampling and Data Collection

Purposive sampling was used to recruit a subsample of men (*n* = 27) who were enrolled in the intervention group through phone calls and tracing techniques. A tracer visited the camps to recruit men who were difficult to reach by phone. This was possible because the researcher had a list of all participants who took a loan and the tracer was very familiar with the areas where these participants lived. Recruitment was stratified across a range of successful completion of the intervention to obtain different opinions of participants with varying levels of success in the program. Participants were purposively selected to achieve representation of men who received all three loans, men who received two loans, and men who failed to pay back their first loan and therefore did not receive any subsequent loans. Men who never received a loan were not included in the qualitative subsample.

Three focus group discussions (FGDs) were conducted by the first author (FM), each group comprising 9 participants for a total of 27 participants. The FGDs were held in a quiet, private room at the parent study’s field office. Sessions were organized near where the participants lived. Light refreshments including juices, water, and sodas were served but no financial incentives were offered. An unstructured approach was used to conduct the FGDs given the exploratory objectives of the study. Each FGD lasted approximately 2 hr and was recorded upon the consent of the participants. Example questions from the FGD guide are as follows: (a) What do you feel are the greatest benefits about participating in the microfinance intervention program? (b) Has your health seeking behavior changed after having participated in this project? The study was ethically approved by University of Dar es salaam Office of the Vice Chancellor (Ref. No: AB3/12B), Regional Administration, and Local Government Office. Written informed consent was obtained from all participants prior to data collection.

### Data Analysis

The FGDs were transcribed and translated from Swahili into English for analysis, which included reviewing of the transcripts multiple times by the research team to inform the development of a codebook. All transcripts were anonymized by the researchers, with any identifying characteristics removed. A directed qualitative analysis approach was used by applying deductive codes based on the FGD guide and existing literature, as well as inductive coding used to identify emerging topics that may have been unexpected. A code summary with quotations was created for each code and used in the second phase of the analysis to generate more specific subtopics related to the inductive and deductive codes. These codes were based on identifying patterns of responses across data from the focus groups and addressing the dimensions of the broader deductive codes (MacQueen et al., 1998). Two independent coders double-coded the data to encourage discussion and refinement of the coding structure (Barbour, 2001). Audio-recorded memos and field notes were also used to enhance reflexivity (Birks et al., 2008). These more focused codes and relevant code combinations became our primary findings. Two of the authors met regularly to establish agreement regarding code definitions, code application, and selection of quotations for illustrative purposes. Illustrative quotations were selected without identifying study participants. When selecting illustrative quotes, we sought to report the direct words of the participants. The code system was then reviewed to identify the primary topics of relevance—those codes that best explained behavior, decision-making, and values—as well as co-occurrences of codes (such as agency and gendered expectations). The Standards for Reporting Qualitative Research was also used to guide the data analysis and reporting of the findings (O’Brien et al., 2014).

## Results

Participants’ characteristics are presented in Table 1. Forty-one percent (*n* = 11) of the men were aged 20–29 and 44% (*n* = 12) of the men were aged 30–39 years. Approximately two thirds (*n* = 18) had a primary education as their highest education level. About half (*n* = 14) of the men were single and 78% (*n* = 21) of the men were daily wage workers. Men discussed multiple benefits of participating in the combined microfinance and peer health education intervention as well as several challenges regarding the microfinance loan amount and accessing general health services. Benefits included increased *agency* and *entrepreneurial activities*. The experience men gained from running their own businesses led to an increase in men’s *employability* and income, which helped to alleviate the stress associated with being unable to financially provide for their families. The perceived benefits, challenges, and recommendations men made for future income generation and health promotion interventions are described in the following text in more detail.

**Table 1.** Characteristics of Participants (*n* = 27).

Participants Characteristics	*n* (%)
Age Groups in Years	
20–24	5 (18.5)
25–29	6 (22.2)
30–34	5 (18.5)
35–39	7 (25.9)
40–44	4 (14.8)
Highest Education Level	
No formal education	3 (11.1)
Primary education	18 (66.7)
Junior high school	4 (14.8)
Senior high school	2 (7.4)
College university	0 (0.0)
Occupation	
Employed in private sector	1 (3.7)
Self-employed	2 (7.4)
Agriculture labor	2 (7.4)
Daily wage	21 (77.8)
Unemployed	1 (3.7)
Marital Status	
Married	6 (22.2)
Single	14 (51.9)
Divorced	4 (14.8)
Widower	3 (11.1)

## Intervention Benefits

### Increased Agency to Test for HIV and Take Out Loans

Participants indicated that they experienced increased *agency* to “check their health” and take out loans due to the intervention. The following participant described how the entrepreneurship education plus peer health education increased his agency and “has taken away my fear” in order to help him take out a loan and even test for HIV.

Basically, I am not very different from my colleagues. The health and entrepreneurship education given during the project has taken away my fear of going to check my health [test for HIV]. Also, it has taken all my fear to take loans in the financial institutions. At first, I was very afraid to take loan. But after I received business knowledge on how to start and run business now, I can take loan at any time I want.

### Stimulating Entrepreneurship Activities

Many men who took out loans were able to use the loans to initiate or increase their *entrepreneurial activities* by starting a new business or strengthening their ongoing businesses. Men’s businesses varied from opening a Tigo Pesa shop, a service that allows people to send and receive money between mobile financial service providers, to selling women’s clothes and coconuts. The following men described how the intervention helped them to start a business:

This program has helped me a lot. Before this program I had no business at all but after I participated in this project, I opened a Tigo Pesa and MPesa shop and until today I have that shop.

I took the loan although it was very small. But I started a business of selling women’s shoes. . . It was a very good business at that time, and I was paying back the loan without a problem. I was selling the shoes.

Some men reported they had experience running small businesses before the intervention as these quotes demonstrate: “I have done a lot of business before entering this project”; “I had business of selling fruits and I am still doing it.” Among men who already had businesses, they reported that their intentions of taking the loans were to strengthen their businesses, “Yes I had business of selling phone accessories and my goal of taking loan was to strengthen this business.” The access to the loans was crucial to some of the men who had ongoing businesses because some of their businesses were not doing well before the intervention as described by the following participant:

Yes, I had a business, and I thank God I was living because of that business. Not that I was getting a big profit, but I lived because of it. I was selling coconuts before entering this project. So I took the loan so as to regain my business that was down.

### Employability as Business Managers

Some men reported that the experience gained from the entrepreneurship training, taking out loans, and becoming business owners in their community helped to increase their employability and build trust in them as potential business managers even though their own business dissolved over time. They were able to gain other employment because people in the community knew that they had entrepreneurial training and experience running their own business. Participants who discussed this theme most often reported that they became a manager for someone else’s businesses as described in the following quote:

Frankly speaking, this project has helped us a lot. . . For instance, myself after I have shown my courage to take loan, although I failed [with the business] . . . there were some people who saw me and they gave me a certain business to run. So those people saw me and believed in me because of this project.

### Mental Health: Increased Agency to Meet Gendered Expectations as Provider

Another benefit that resulted from the businesses men started or strengthened with the loans was the extra income they gained that helped to increase their agency and alleviate the stress associated with taking care of their families. As fathers and husbands, men were sometimes the sole providers for their households, as one man stated, “We are everything to our families.” The challenges and stress associated with unemployment and inability to gain income to pay for their families’ expenses can negatively affect men’s mental health. “We need to find money for our family to eat, children to go to school etc,” said one man as he described the need for him to secure employment or business opportunities in order to make enough money to provide for his family. The income from the businesses may have helped reduce men’s stressors by increasing their agency to provide for their families as indicated by one man, “I am getting small but its mine and I can pay for my children school’s fees and transport and my family is living because of this business.” Another man who did not have a family before the intervention and used the loan to strengthen his business reported that he decided to start a family because of the extra income from his business:

Myself, I got married and now I have a wife and a kid. This is after I took the loan and strengthened my business and things went well to the point I decided to be with someone who can help me and make family.

## Remaining Challenges and Recommendations

### General Health Services

Though HIV testing uptake was higher among men in the intervention, they reported that their general health–seeking behaviors were not influenced by their participation in the microfinance and peer health leadership intervention. Instead, men highlighted multiple challenges that continued to inhibit use of general health services for consideration in future health interventions targeting men. Men reported health-care-seeking challenges such as *cost of services* and *family expectations* that required men to spend time looking for employment or earning money, instead of using their time to commute and wait in queues (or lines) at health facilities. Even though some men experienced increased income, most were still unable to afford regular for-pay health-care services. They described multifaceted barriers to accessing care, even though most men reported being internally motivated to do so.

To go to the health facilities just to check our health is impossible because of the high cost of health services. Diseases that you can check for free is only HIV/AIDS and TB but other checkups you need money and it’s a lot of money. So we are struggling even to get money to buy food then you want to use a small amount we got to just go to check our health while we do not feel sick? My friends in here we do not have that attitude.

Men also reported that the time they needed to spend earning money in order to provide for their families presented unique challenges to seeking health services. Men prioritized their time and money on income generation activities as opposed to health services because they were responsible for financially providing for their families. Time-related barriers had the greatest impact on men’s use of preventative services, whereby men were expected to attend routine checkups when they still felt healthy. Preventive care was seen as important, but a direct threat to men’s income because it reduced valuable income-earning time. When men had to choose between the immediate benefits of daily income and the potential long-term benefits of general health checkups, many noted that immediate income for their families was the obvious choice.

Yeah there is a great advantage and need for men to participate in these health projects, but our time is very limited, my brother. If you miss a business opportunity while engaging yourself these health issue, who is going to provide for your family? We want so bad to participate because we know the importance of these health projects, but we let our wives to participate and us men we go to find money for daily living.

In response to the costs associated with seeking health services, a few participants suggested that health insurance be incorporated into future interventions. These participants believed health insurance would alleviate high costs associated with health services and make it easier for men to attend routine screening services other than HIV testing.

My brother, the health services are very high in our public hospitals. We really need a project of health insurance in our camps. Most of us we could not afford the costs of paying the health services and that is why in our areas no one has the habit of just going to the hospitals to check for his health. So, please, if you guys can come up with a project that has a component of health insurance we will really appreciate [it], and we will fully participate in that program.

We want a project that would allow us to get National Health Insurance, we are ready to contribute in this project [an insurance plan]. Health service [costs] are too high in our country, so if we get a National Health Insurance we can pay for it [services].

### Small Microloans and Difficult Dynamics Within Microfinance Groups

Despite the benefits associated with the intervention, participants reported a few challenges associated with the microfinance component of the intervention that limited their entrepreneurial potential and actual income. While some men were able to start and sustain their businesses, others reported that the loan amount was not enough to develop or support their businesses. “I wanted to do big business for which the loan we got was too small. . . so we ended up eating all money.” Another man who started a business reported, “I started a business but because of a small capital we received I could not make it far.” One man described how the small loan amount would be better for people who already have a business as opposed to starting a business:

Our goals were to succeed in life, but we could not achieve our goals because the opportunity was very small. . . I could not even start a business. You cannot start a business. Maybe if you had a business and you took the loan to strengthen your business. I wanted to sell mitumba [secondhand clothes] but I could not make it because the loan was small.

The group structure requirement for the loan also made it difficult for individual men to succeed if their group was unreliable. The failure to start or succeed in their businesses prevented men from repaying the first loan. The inability of group members to repay the first loan affected other group members’ ability of being eligible to receive the second ($185) and third ($285) loans even if a few individual members had repaid:

I did not finish all the phases of the loan and this was caused by my colleagues in the group. The idea to give us the loan in group was very bad and it failed us. There are some people who did not attend the entrepreneurship training and they were given the loan so these people troubled us my friend. I wanted to repay my loan quickly so as to take another loan but you find other people in a group did not want to repay their loan, and you cannot go to YOSEFO yourself to ask for another loan while your members did not finish to repay their loan. So this made me very angry and I withdraw myself from the first loan.

In response to the microfinance challenges, some participants recommended that loan amounts be based on type of businesses instead of a static amount for everyone regardless of the business idea. They also recommended having monthly repayments instead of weekly repayments in order to give them time to earn the necessary income needed; some businesses take time to earn a profit since several weeks may have a small or negative balance based on the natural ebbs and flows of small businesses.

The loan should be given according to the business proposal of the person who need the loan. If someone wants to construct an industry, then he should be given the amount that’s enough to construct the industry. So the loan should be given according to the business proposal.

The loan repayment should be at least one month not in a week. A week is very small and troublesome for us to go to the YOSEFO and repay the loan. At least a month would be a good time for loan repayment.

Additional income-generating recommendations included having a sponsor who can finance and provide the equipment for the business. This idea is aligned with the experience some men reported regarding how their employability as business managers increased due to other people trusting them because of their participation in the entrepreneurship training and microfinance program. Similarly, men could receive the training and be hired to run a business developed by the intervention team or matched with employers if they wanted to avoid the challenges of repaying for a loan. The following quote supports this income-generating view without the use of microfinance being given to men directly:

I also think you can design a project to provide equipment’s and not money, a person who needs to borrow comes with his plan that he wants to do a certain business so you as an organization you open it the business and hand over to him for running it. For instance, you can open a barbershop for me and I do the business and every month I pay the interest.

## Discussion

Overall, men reported several benefits from participating in a combined microfinance and peer health leadership intervention, which included increased agency to take out loans and increased stimulation of their entrepreneurial activities. One unexpected benefit was the increased employability, which led to men receiving business management opportunities even though they were not successful in their own businesses. In addition, the income generated from their businesses allowed men to meet expectations as providers for their families, thereby improving their social and mental well-being. The increased agency men experienced also encouraged them to check their health by testing for HIV. This is supported by the quantitative data in the parent study showing that men in the intervention group were more likely to have tested for HIV than their counterparts in the control group (Maman et al., 2020). In contrast, men also experienced multiple challenges, some related to their income generation activities and others related to general health–care-seeking behavior at local health facilities. Men recommended increased loan size and health and social insurance to address these challenges and enhance future interventions designed to provide socioeconomic opportunities and promote health-seeking behaviors among men in the region.

The increased agency men experienced that motivated them to take out loans to either start a new business or strengthen an existing business is supported by other studies reporting that entrepreneurship training can increase agency by improving business knowledge (Karlan & Valdivia, 2011) competencies and intention toward self-employment (Sánchez, 2013). The businesses men started or strengthened created extra income for some men, thereby reducing the economic stressors associated with underemployment and expectations as breadwinners for their families. These stressors are linked with anxiety and depression among men in Tanzania and elsewhere (Hamad et al., 2008; Hill et al., 2018). Therefore, the main pathway through which the intervention improved men’s health was through the increased income generated from their businesses that helped reduce the financial stressors men face as breadwinners for their families, which can negatively affect their mental health (Hamad et al., 2008; Silberschmidt, 2001). Men reported that they were *everything* for their families and *needed to find money* to pay the fees for their children’s school and other expenses and described how the profits from their businesses allowed them to accomplish these goals and even encouraged one man to start a family.

Not all men were able to benefit similarly, and a number of men reported that they were not able to achieve their goals as providers for themselves or their families. Other studies have reported that entrepreneurship training and loans with low interests rates alone were not sufficient to ensure that all participants were ready to take a loan, start a business, and become successful entrepreneurs (Oosterbeek et al., 2010; Premand et al., 2016). The challenges that hindered them from succeeding included the small loan amount and group structure for the loan application, paralleling some of the contextual, structural, and individual factors described in an earlier study that influenced men to proceed from training to apply for a loan, establish a business, and repay the loan (Balvanz et al., 2019). To address these challenges, men recommended increasing the loan amounts and providing loans to individuals instead of groups, which in turn may help increase the number of men who take out loans and ultimately enhance the likelihood that their business succeed and they are able to repay their loan.

The increased agency men experienced to check their health (i.e., HIV testing) is consistent with previous studies that also provided entrepreneurship training and health education (Lorenzetti et al., 2017; Sánchez, 2013). Some men reported that the increased health knowledge reduced their fears and motivated them to go “check their health.” “Checking” one’s health can have several meaning but based on a previous qualitative study conducted with the same group of men, checking one’s health refers to seeking HIV testing and served as non-stigmatizing way to speak about HIV testing among group members (Conserve et al., 2018). A systematic review of integrated microfinance and health education interventions found a positive effect on health knowledge and behaviors, but not health status (Lorenzetti et al., 2017). Findings also revealed that men would like to seek health services other than HIV testing, which is free, but the required cost and time away from work associated with visiting health facilities prevented them from doing so (Mamdani & Bangser, 2004). It is possible that if the men had been microfinance clients for a longer period of time they may have accrued enough resources to afford routine health services. In a study with women that found a positive association between microfinance participation and health-seeking behaviors (i.e., antenatal care), there was a stronger association with health-care seeking as duration of participation in the microfinance program increased (Bhuiya et al., 2018). Further studies for on male clients of microfinance should explore if similar health-seeking behavior exists among men who participate in microfinance programs for a longer period of time.

To address challenges to general health–care-seeking behavior, men made several recommendations that can be incorporated into future microfinance interventions. The first recommendation was to incorporate a health insurance component, which is consistent with the approach several MFIs have used in other countries including, but not limited to, Benin, Bolivia, Burkina Faso, India, and the Philippines (Reinsch et al., 2011). Health protection services offered by some MFIs vary from health micro-insurance to linkages to health providers and access to health products (Lorenzetti et al., 2017). MFIs in Tanzania have also piloted and implemented different micro health insurance programs and experiments with limited documentation of their impact (Janssens & Kramer, 2016; McCord, 2001). More research is needed in Tanzania to determine what challenges are associated with incorporating micro health insurance into MFI programs and what barriers and facilitators of enrollment into micro health insurance exist. Another option is to increase awareness of other health insurance programs available in the country such as the National Health Insurance Fund and the Community Health Fund for those who can afford to enroll in these schemes and help identify male-friendly health clinics supported by donors, which may provide additional services for free for those who are unable to enroll in health insurances programs. Another solution from HIV research is to develop more male-friendly health service delivery strategies. The design and delivery of health service programs in the region primarily focus on women and children, which may unintentionally deter men from utilizing health-care services (Peacock et al., 2009; Yeatman et al., 2018). Health services that are designed with men in mind, and actively encourage men’s engagement, may help overcome the financial barriers men face by reducing wait times and creating positive experiences for men that may outweigh the financial costs associated with care.

## Limitations

While this is one of the first studies to explore perceived health and entrepreneurial benefits and challenges among men who participated in a combined health and microfinance intervention, there are some limitations that merit consideration. First, the analysis relied on a small sample of men from one program in Dar es Salaam, Tanzania. Findings may not be representative of all men who participated in the parent study and may not be generalizable to other settings. Second, only men who received one or more loans were included. Findings do not reflect perceived benefits and challenges of men who were unable to take loans—these men may be qualitatively different than men who received microloans, and their experiences with the combined health and microfinance intervention may also differ. Despite these limitations, this study presents lessons learned from designing and implementing economic-strengthening interventions to promote health-seeking behaviors among men in resource-constrained settings. The findings of this study can inform future research in similar contexts.

## Conclusion

Men in low-resource settings in Tanzania perceived notable benefits from participation in a combined microfinance and peer health leadership intervention, particularly increased knowledge, ability to navigate business situations, and improved mental health as a result of improved financial stability. However, men also faced challenges that prevented them from accessing general health services, succeeding in their businesses, and repaying their loans. Challenges may be addressed by increasing loan amounts, providing individual rather than group loans, and incorporating health insurance into programs.
